# Comprehensive *N*-glycosylation mapping of envelope glycoprotein from tick-borne encephalitis virus grown in human and tick cells

**DOI:** 10.1038/s41598-020-70082-2

**Published:** 2020-08-06

**Authors:** Erika Lattová, Petra Straková, Petra Pokorná-Formanová, Libor Grubhoffer, Lesley Bell-Sakyi, Zbyněk Zdráhal, Martin Palus, Daniel Ruzek

**Affiliations:** 1 Central European Institute of Technology, Masaryk University, Kamenice 5, 62500 Brno, Czech Republic; 2 Veterinary Research Institute, Hudcova 296/70, 62100 Brno, Czech Republic; 3 Institute of Parasitology, Biology Centre of the Czech Academy of Sciences, Branisovska 31, 37005 Ceske Budejovice, Czech Republic; 4 Department of Infection Biology and Microbiome, Institute of Infection, Ecological and Veterinary Sciences, University of Liverpool, 146 Brownlow Hill, Liverpool, L3 5RF UK; 5 National Centre for Biomolecular Research, Faculty of Science, Masaryk University, Kamenice 5, 62500 Brno, Czech Republic

**Keywords:** Virology, Glycobiology

## Abstract

Tick-borne encephalitis virus (TBEV) is the causative agent of severe human neuroinfections that most commonly occur after a tick bite. *N*-Glycosylation of the TBEV envelope (E) glycoprotein is critical for virus egress in mammalian cells, but not in tick cells. In addition, glycans have been reported to mask specific antigenic sites from recognition by neutralizing antibodies. In this regard, the main purpose of our study was to investigate the profile of *N*-glycans linked to the E protein of TBEV when grown in human neuronal cells and compare it to the profile of virus grown in tick cells. Mass spectrometric analysis revealed significant differences in these profiles. High-mannose glycan with five mannose residues (*Man*_*5*_*GlcNAc*_*2*_), a complex biantennary galactosylated structure with core fucose (*Gal*_*2*_*GlcNAc*_*2*_*Man*_*3*_*GlcNAc*_*2*_*Fuc*), and a group of hybrid glycans with the composition *Gal*_*0-1*_*GlcNAc*_*1*_*Man*_*3-5*_*GlcNAc*_*2*_*Fuc*_*0-1*_ were confirmed as the main asparagine-linked oligosaccharides on the surface of TBEV derived from human neuronal cells. The observed pattern was supported by examination of the glycopeptides, providing additional information about the glycosylation site in the E protein. In contrast, the profile of TBEV grown in tick cells showed that paucimannose (*Man*_*3-4*_* GlcNAc*_*2*_*Fuc*_*0-1*_) and high-mannose structures with five and six mannoses (*Man*_*5-6*_*GlcNAc*_*2*_) were major glycans on the viral surface. The reported results complement existing crystallography and cryoelectron tomography data on the E protein structure and could be instrumental for designing carbohydrate-binding antiviral agents active against TBEV.

## Introduction

Tick-borne encephalitis (TBE) is one of the most severe viral diseases of the central nervous system in humans^1^. The causative agent, tick-borne encephalitis virus (TBEV), has expanded to new geographical areas, and several thousand new cases of human TBE are reported in European and Asian countries each year. TBEV belongs to the family *Flaviviridae* and the genus *Flavivirus* that includes over 70 viruses, many of which are important human pathogens^2,3^. Infectious TBEV virions are small spherical particles, about 50 nm in diameter, containing a positive-sense, single-stranded RNA genome forming, together with the viral capsid protein, a nucleocapsid core^4^. This nucleocapsid is surrounded by a lipid bilayer, which incorporates the E glycoprotein and membrane glycoprotein (M). Two E-proteins and two M-proteins form a compact heterotetramer, which is the basic building block of the mature virion^5^.

E glycoprotein is the only viral protein exposed on the surface of the mature virion^6,7^. It has at least two essential roles during the virus life cycle—the protein mediates interaction of the virus particle with a receptor at the cytoplasmic membrane of the host cell, and mediates membrane fusion of the viral envelope with an endosomal membrane after cellular uptake by receptor-mediated endocytosis^8^. It is also the main antigenic determinant of the virus and main inducer of immune responses in the infected mammalian host. It is also recognized that E protein is functionally important as a crucial determinant of TBEV virulence^9^.

TBEV E protein has one *N*-linked glycosylation site at Asn154^10^. It is believed that proper folding of the E protein and stabilization of dimer contacts between two E molecules, its interaction with cell receptors and its immunogenicity can be largely influenced by asparagine-linked oligosaccharides^11^. In addition, most of the available data indicate that flavivirus E protein glycosylation is associated with increased infectivity and plays an important role in virion assembly, tropism and virulence/pathogenesis^12–16^. Interestingly, in the case of TBEV, virus infectivity was not affected after deglycosylation^17^. However, deletion of glycans affected the conformation of E protein during TBEV maturation in mammalian cells and reduced TBEV virulence in mice^14^. On the other hand, deletion of the glycosylation site of the E protein had no effect on the growth of TBEV in tick cells. This may be associated with different TBEV maturation processes seen in mammalian and tick cells^18^. Data on other flaviviruses indicate that virions produced in the invertebrate vector and mammalian cells may differ in oligosaccharide compositions, as invertebrate and mammalian cells process glycans differently during exocytosis^19,20^. For example, using lectin blots it was found that dengue virus grown in mosquito cells contained two glycans, one paucimannose and one with high-mannose composition, while one high-mannose and one complex glycan were identified when the virus was grown in mammalian cells^20^.

Despite the fact that TBEV E protein has been characterized extensively, including determination of its structure using X-ray crystallography^21^ and cryoelectron tomography (cryo-EM)^22^^,^ the precise compositions of *N-*glycans attached to TBEV E protein derived from mammalian and tick hosts remain unknown. The only available study partially characterized glycans of TBEV grown in primary chick embryo cells via lectin chemistry, and the results indicated heterogeneity in the glycosylation of E proteins^23^. Since the glycosylation pattern can have a large impact on virus properties, including pathogenicity and virulence^10^^,^ this needs to be further addressed.

In this study, we report in-depth analysis of asparagine-linked oligosaccharides of mature TBEV derived from mammalian and tick cells. To validate observed differences in profiles, we similarly evaluated *N*-glycans of uninfected neuroblastoma and tick cells. To the best of our knowledge, this is the first report that provides evidence that the carbohydrate profile of TBEV E protein differs based on the type of host cells, and complements existing crystallography and cryo-EM data on the TBEV E glycoprotein structure.

## Materials and methods

### Statement

All methods were carried out in accordance with relevant guidelines. All work with infectious samples was done in an accredited BSL-3 facility at the Veterinary Research Institute in Brno and the Institute of Parasitology, Biology Centre of the Czech Academy of Sciences in Ceske Budejovice, Czech Republic. The use of TBEV in the experiments was approved by the Department of Biological Weapons Prohibition of the State Office for Nuclear Protection in Prague, Czech Republic (Permit No. SÚJB/OKZBZ/15978/2013).

### Virus and cells

Low-passage TBEV strain Hypr (originally isolated from the blood of a deceased 10-year-old child with TBE in Moravia, Czechoslovakia^24^^,^ was provided by the Collection of Arboviruses, Biology Centre of the Czech Academy of Sciences. Human neuroblastoma cells UKF-NB4^25^ were grown in Iscove’s modified Dulbecco’s medium (Biosera) supplemented with 10% FBS at 37 °C in the presence of 5% CO_2_. The *Ixodes ricinus* tick cell line IRE/CTVM19^26^ was grown at 28 °C in L-15 (Leibovitz) medium supplemented with 10% tryptose phosphate broth (Gibco), 20% FBS (Biosera), 2 mM L-glutamine, 100 μg/ml penicillin and 100 μg/ml streptomycin (Sigma-Aldrich). For virus production, the same media but without FBS were used.

### Virus infection and purification

UKF-NB4 cells grown to 100% confluence in 15 flasks, each with a growth area of 300 cm^2^ (T300), were infected with TBEV at a multiplicity of infection (MOI) of 0.5. After 24 h of incubation at 37 °C, the medium was replaced with fresh medium without FBS (to minimize residual contamination with FBS glycoproteins in subsequent analyses). The culture media were harvested 35 h post infection and clarified by centrifugation at 5,700 × *g* for 10 min at 4 °C as described previously^22^. The supernatant was precipitated by adding PEG 8000 (Sigma-Aldrich) to a final concentration of 8% (w/v) and incubating overnight at 9 °C with gentle shaking (130 rpm).^22^ After that, the virus was pelleted by centrifugation at 10,500 × *g* for 50 min at 4 °C. The resulting pellet was resuspended in 2 ml of NTE buffer (20 mM Tris, 120 mM NaCl, 1 mM EDTA, pH 8.5^22^. The solution was clarified by centrifugation at 1,500 × g for 5 min at 4 °C. The solution was loaded onto a step tartrate gradient (10, 15, 20, 25, 30, and 35% K_2_C_4_H_4_O_6_ in NTE buffer). After separation in a Himac CP80WX ultracentrifuge (Hitachi) with a P40ST swinging bucket rotor at 32,000 rpm for 2 h at 4 °C, a visible band containing the virus was harvested using a syringe with a needle (20G)^22^. Finally, the collected virus was repeatedly diluted with 4 ml of NTE buffer and concentrated to a final volume of 100 µl using a centrifugal filter concentrator with a 100 kDa cut-off (Vivaspin 6 Centrifugal Concentrator, Vivaproducts)^22^.

IRE/CTVM19 cells grown in 15–20 flat-sided cell culture tubes (Nunc) were infected with TBEV strain Hypr at a MOI of 5, as described previously^27^. After 24 h, cell culture medium containing FBS was replaced with serum-free medium and harvested at 7–9 days post infection. The virus was precipitated by adding PEG 8000 to a final concentration of 8% (w/v) and incubating overnight at 4 °C with gentle shaking. After that, the virus was pelleted by centrifugation at 10,500 × g for 50 min at 4 °C. The resulting pellet was resuspended in 2 ml of NTE buffer, repeatedly diluted with 3 ml of NTE buffer and concentrated to a final volume of 100 µl using the centrifugal filter concentrator with a 100-kDa cut off. Afterwards, all samples were subjected to Western blot analysis and SDS-PAGE gel electrophoresis or immediately processed for mass spectrometric (MS) analysis.

### Preparation of excised gel bands for digestion in situ

After SDS-PAGE separation, excised gel bands were cut into smaller pieces (~ 1 mm^2^), transferred into Eppendorf tubes (2 mL) and processed using a series of washing steps with 20 mM NH_4_HCO_3_ (AB; 2 × 150 μL), a mixture of acetonitrile (ACN) and AB (1:1; 2 × 150 μL), and ACN (2 × 150 μL). Then gels were dried in a SpeedVac centrifuge to remove solvent. Dried gel pieces were rehydrated in 10 mM AB (100 μL) and prepared for digestion as described below.

### Release of *N*-glycans

The rehydrated gel pieces containing purified TBEV E protein were incubated with PNGase F (3 mU, Asparia Glycomics) at 37 °C for 3 h, followed by sonication (5 min) to enrich the supernatant with released oligosaccharides.

Uninfected UKF-NB4 and IRE/CTVM19 cells were first washed with 70% ethanol (100 μL) and after 5 min of sonication, the ethanol solution was evaporated under vacuum. Then the samples underwent extraction as described previously^28^. Briefly, chloroform–methanol-water solution in ratio 8:4:1 (~ 300 µL) was added to each sample and sonicated for 5 min. After centrifugation (5 min at 3,000 × g), supernatants were discarded and traces of organic solvent were removed from pellets by evaporation under vacuum. Dried pellets were resuspended in 5 mM AB and incubated with PNGase F for 3 h.

Half of the each PNGase F digested mixture was further incubated with neuraminidase (*Clostridium perfringens*, Sigma) at 37 °C for 2 h to remove sialic acids. Prior to purification, all digests were analyzed for the presence of *N*-glycans by applying on-target labeling with phenylhydrazine (PHN) according to the protocol described in the section on MS.

### SPE purification

After enzymatic digestion, supernatants with released *N*-glycans were purified on STRATA-X-C (33um Polymeric Strong Cation, 10 mg/1 mL) and graphitized carbon (1 ml tubes, Supelco) cartridges. The XC column was washed with 100% ACN (5 × 800 μL) and then with deionized water (5 × 800 μL). The PNGaseF digest (40 μL) was applied onto the surface of the cartridge, left to penetrate into the carrier, and then washed with deionized water (3 × 50 µL). From the first appearance of eluate from the cartridge, the eluates were collected and analyzed. When using the digest from whole cell lysates, the XC-eluates were immediately loaded onto graphitized carbon cartridges (1 ml tubes, Supelco) and the same protocol was followed as described previously^29^. Glycan fractions were combined, evaporated and analyzed by MALDI-MS. In the case of glycan pools obtained after purification of samples digested only with PNGase F, permethylation was applied as described below.

### Permethylation

Dried *N*-glycan pools were permethylated according to a published protocol^30^, with slight modifications. Briefly, dimethyl sulfoxide (50 μL), powdered NaOH (~ 1 mg) and methyl iodide (2 × 15 μL) were added to the tube with dried glycans and vortexed at room temperature for 40 min. The reaction was stopped by dipping the tube with the reaction mixture into liquid nitrogen (~ 30 s), followed by addition of deionized water (300 μL), and permethylated glycans were extracted with CHCl_3_ (300 μL). The chloroform extract was washed with deionized water (~ 4 × 300 μL), evaporated and dissolved in 60% methanol (10 μL) prior to MS analysis.

### In-gel trypsin digestion and HPLC-fractionation

Rehydrated gel pieces (25 mM AB) were digested with trypsin (Promega) at 37 °C for 4 h. After short sonication (3 min) and centrifugation (2,000 × *g* for 3 min), the supernatant was pipetted into a new tube, evaporated and dissolved in deionized water (50 μL). The tryptic digest (10 μL) was fractionated on a Dionex UltiMate 3000 XRS (Thermo Scientific) system coupled with a Vydac C18 HPLC column (218 TP54). Solvent A was deionized water with 0.1% trifluoroacetic acid (TFA) and solvent B consisted of 80% ACN with 0.1% TFA. An elution gradient was applied from 5 to 80% ACN over 40 min with flow rate 0.5 mL/min. Fractions were collected after each 0.5 min, then completely dried, resuspended in deionized water (10 μL) and analyzed by MALDI-MS as described below.

### Mass spectrometric analysis (MS)

MALDI-MS analysis was performed on an UltrafleXtreme mass spectrometer furnished with a Smartbeam-II laser and LIFT technology (Bruker, Germany). The instrument was used in the reflectron positive ion mode, calibrated externally using a mixture of known peptides over a mass range of 800–8,000 Da. Individual precursor ions were manually selected for LIFT (MS/MS) experiments. The choice of matrix depended on the type of analyzed sample. For native *N*-glycans, the on-target derivatization was applied^31^. Briefly, matrix was prepared by mixing 6-aza-2-thiothymine and phenylhydrazine hydrochloride (ATT/PHN.HCl, 2:1) with a final concentration of 3% in ACN/deionized water (1:1). A 0.8 μL aliquot of this solution was spotted on the surface of an AnchorChip target, immediately followed by 1.5 μL of PNGase F digest or purified glycan fraction. To the still wet spot, 0.6 μL of phenylhydrazine (PHN) labeling reagent (PHN:ACN:water in ratio 1:1:4) was added and left to air dry (5–10 min). The MS and MS/MS spectra of *N*-glycans were evaluated manually using fragmentation rules described previously^31^. Exoglycosidase cleavages were performed on desialylated pools with galactosidase (bovine testes, Sigma), α-mannosidase and β-N-acetylglucosaminidase (Jack beans,Sigma) according to the protocol supplied by the manufacturer, and were used to confirm monosaccharide identity. In MS spectra, glycan peaks were annotated with structures using the symbolic nomenclature proposed by the Consortium for Functional Glycomics (https://www.functionalglycomics.org). The assignment of fragment ions in MS/MS spectra was based on the Domon & Costello nomenclature^32^.

For MS analysis of permethylated glycans and HPLC-fractions of the tryptic digest, 2,5-dihydroxybenzoic acid (DHB, Sigma) dissolved in 50% ACN solution was used as a matrix. Glycopeptides were identified manually according to characteristic differences corresponding to monosaccharide residues and then confirmed by MS/MS analysis. The assignment of oligosaccharide structure on the peptides was based on a combination of results acquired from tandem MS analysis of both glycopeptides and *N*-glycans cleaved from intact protein. The compositions of glycosylated peptide backbones were next confirmed by LIFT analysis of peptides after deglycosylation with PNGase F. Peptide identities based on the MS/MS data were manually assigned using the nomenclature of *Roepstorf & Fohlman*^33^, and then verified using the Mascot search engine (www.matrixscience.com) with an error tolerance on the monoisotopic ions of up to 0.6 Da. Database searches were conducted with no fixed modification and variable modifications and the query was made against taxonomy “All entries”. In addition, BLASTp searches for manually-assigned peptide sequences were performed (https://blast.ncbi.nlm.nih.gov/Blast.cgi).

For graphical illustration of *N*-glycan differences between samples, the spectra were processed using the FlexAnalysis software (Bruker) and evaluated manually. In MS spectrum recorded after a desialylation, the acidic glycans were assigned based on detection of new peaks or increased intensities when compared with a spectrum recorded prior to an incubation with neuraminidase. At least two neutral glycans of the same composition detected in both spectra were used as an internal standard. The type and number of sialic residue(s) were confirmed after permethylation of purified PNGase F digests. The relative abundances (r.a.) of individual glycans were calculated from their normalized intensities using Microsoft Excel.

## Results

In this study, we employed the MALDI-MS technique to thoroughly investigate *N*-glycans of the E glycoprotein isolated from TBEV grown in human (neuroblastoma) and tick (IRE/CTVM19) cells, and *N*-glycans from uninfected cells of the same types. Oligosaccharides were analyzed after deglycosylation with PNGase F and compared with the same samples following desialylation with neuraminidase. MALDI-MS and MS/MS analysis was performed on PHN-labeled oligosaccharides^31^. The presence and type of sialic acid was also validated by examination of glycans after permethylation. The combination of different preparative approaches was useful in the identification of a higher number of glycans. All *N*-glycans identified in the examined samples are listed in Supplementary Table 1. In the case of TBEV grown in neuronal cells, the purified E glycoprotein additionally underwent digestion with trypsin, and isolated glycopeptides were evaluated to validate the glycosylation pattern and confirm a glycosylation site in the protein. A flowchart illustrating the employment of preparation procedures used for investigation of *N*-glycosylation is shown in Supplementary Scheme 1.

### *N*-Glycosylation of TBEV grown in human neuroblastoma cells

#### *N*-Glycan analysis

**Figure 1:**
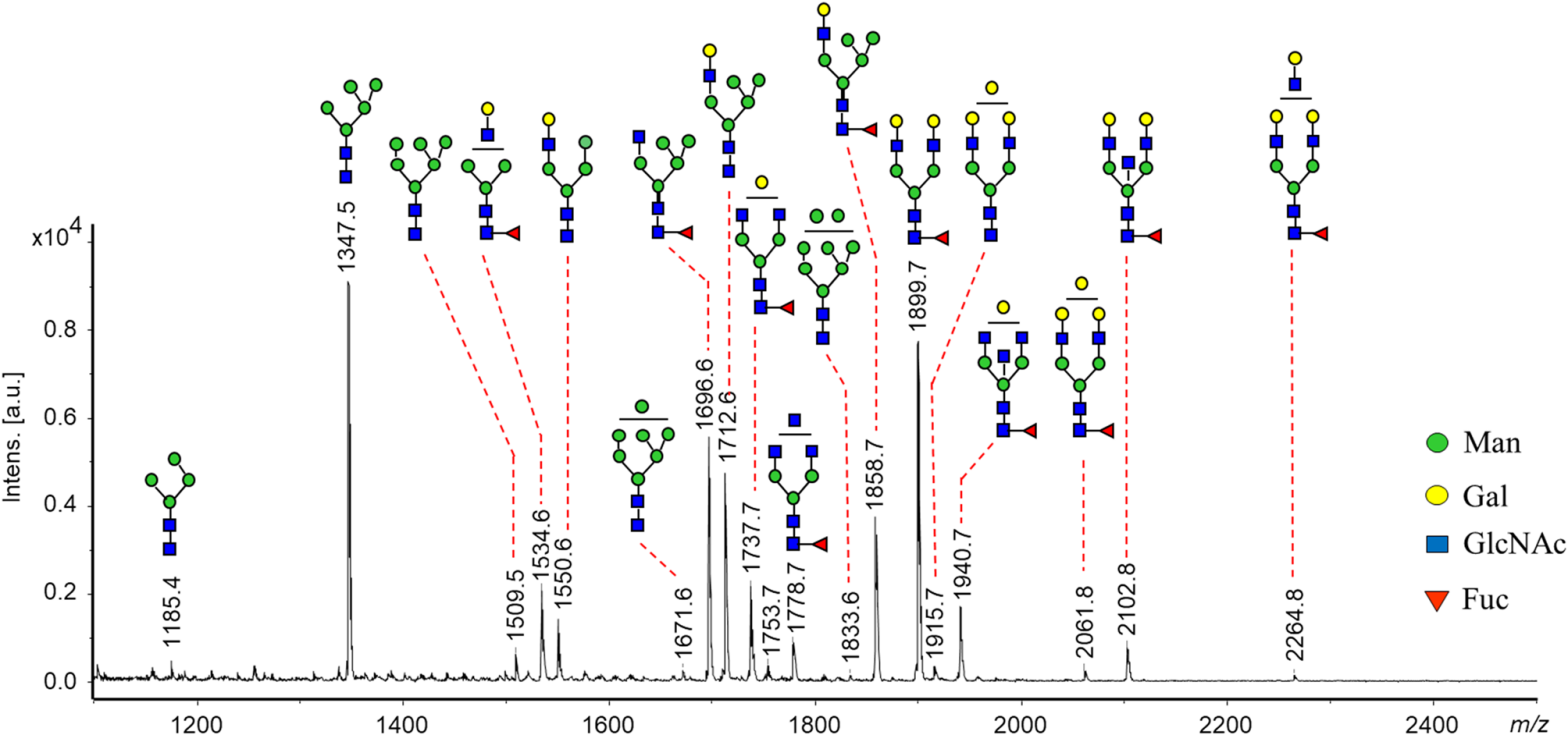
MALDI-MS spectrum of *N*-glycans recorded from SDS-purified E glycoprotein of TBEV grown in human neuroblastoma cells (UKF-NB4). Glycans were detected immediately after incubation with PNGase F and neuraminidase applying on-target derivatization with PHN (+ 90.06 increase in mass and detected as MNa^+^ ions). No purification procedure was applied here.

MS analysis of glycans released from intact SDS-purified E glycoprotein (Supplementary Fig. 1) indicated that neutral oligosaccharides were major structures and corresponded to compositions of high-mannose, hybrid and complex biantennary oligosaccharides including their fucosylated analogs (Fig. 1). Representative tandem mass spectra recorded from detected glycan peaks are shown in Supplementary Fig. 2. High-mannose glycans with compositions *Man*_*5-8*_*GlcNAc*_*2*_ were detected at *m/z* 1347.5, 1509.5, 1671.6 and 1833.6 (Fig. 1). Among them, only the structure with five mannose residues (*Man*_*5*_*GlcNAc*_*2*_; *m/z* 1347.5) was observed with the highest intensity (~ 25%). The MS/MS fragmentation pattern of this glycan supported the prevalence of an isomer with no additional residue on the 3-linked mannose since the loss of 3-positioned hexose from the B and C fragment ions resulted in the production of abundant B_3_/Y_3β_ ions (Supplementary Fig. 2A, *m/z* 671).

**Figure 2:**
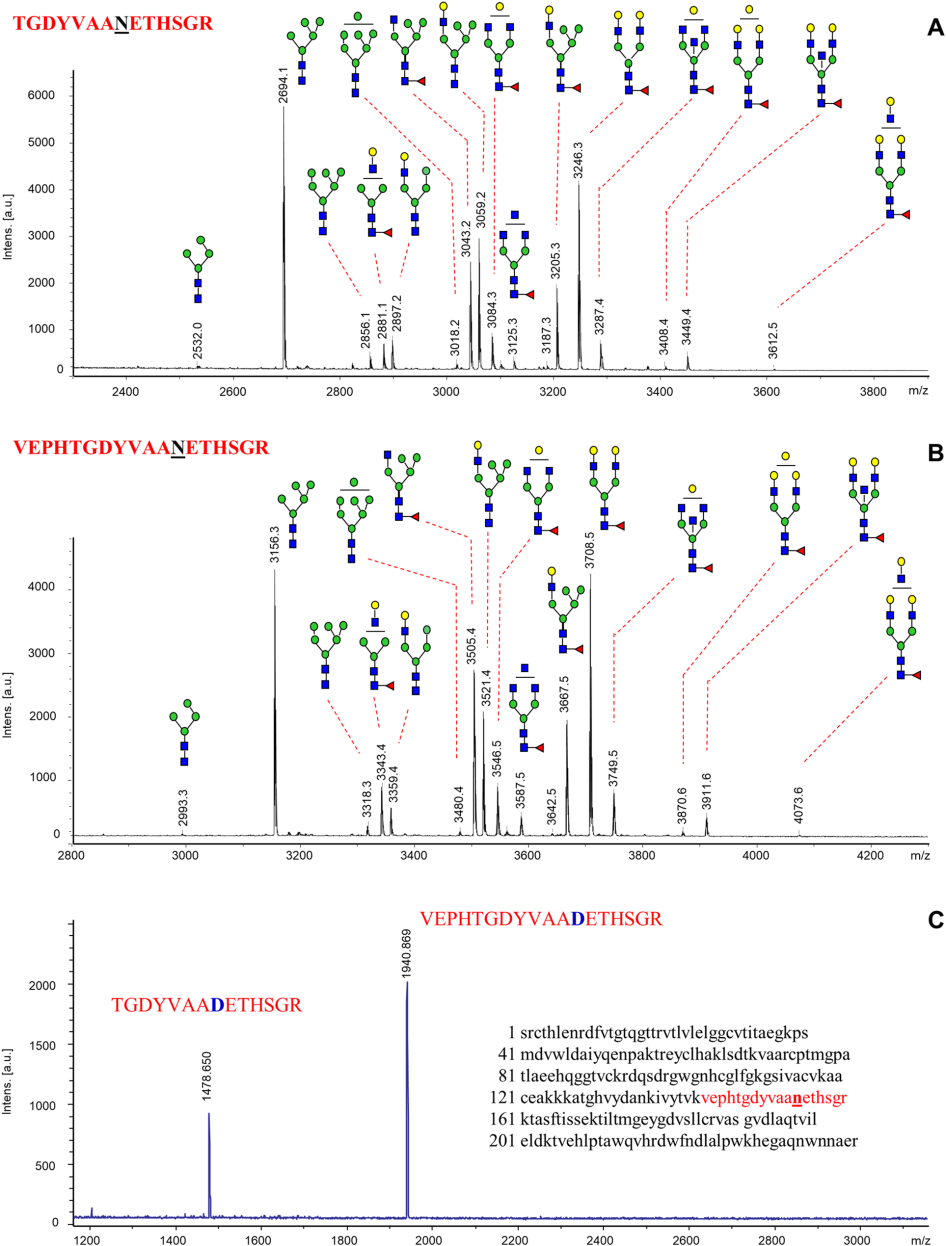
MALDI-MS spectra of glycopeptides analyzed in the E glycoprotein of TBEV grown in human neuroblastoma cells (UKF-NB4). Glycopeptides were obtained after in-gel trypsin digestion and RP-HPLC fractionation: (**A**) fraction with elution time 13–14 min; (**B**) fraction eluted at 16–18 min; and (**C**) MS spectrum of deglycosylated peptides recorded from fraction with elution time 15 min after incubation with PNGase F; inset shows peptide sequence suggested in TBEV^34^. All peaks are as [M + H]^+^. Additional details are shown in Supplementary Figs. 3 and 4.

Hybrid glycans were observed at *m/z* 1534.6 (*Gal*_*1*_*GlcNAc*_*1*_*Man*_*3*_*GlcNAc*_*2*_*Fuc*) and 1550.6 *(Gal*_*1*_*GlcNAc*_*1*_*Man*_*4*_*GlcNAc*_*2*_), and those with higher incidence were detected at *m/z* 1696.6, 1712.6 and 1858.7 (~ 30%). The MS/MS analysis supported the prevalence of the isomers as assigned for each peak in Fig. 1 (for more details see fragmentation pattern in Supplementary Fig. 2B). The other abundant glycan (~ 20%) was observed at *m/z* 1899.7 and matched to the complex biantennary digalactosylated glycan with fucose linked on the reducing termini—*Gal*_*2*_*GlcNAc*_*2*_*Man*_*3*_*GlcNAc*_*2*_*Fuc* (Fig. 1). The fragmentation pattern provided no evidence for presence of isomer with fucose on the antenna (Supplementary Fig. 2C). The other core fucosylated complex glycans observed with lower abundances corresponded to compositions *Gal*_*1*_*GlcNAc*_*2*_*Man*_*3*_*GlcNAc*_*2*_*Fuc* (*m/z* 1737.7) and *GlcNAc*_*3*_*Man*_*3*_*GlcNAc*_*2*_*Fuc* (*m/z* 1778.7). Peaks detected at *m/z* 1940.7 and 2102.8 under tandem MS conditions produced the fragment ions supporting the structures of core fucosylated glycans with compositions *Gal*_*1-2*_*GlcNAc*_*3*_*Man*_*3*_*GlcNAc*_*2*_*Fuc* in favor of an isomer with bisecting moiety^31^.

Sialylated glycans bore *N*-acetylneuraminic (NeuAc*)* and smaller amounts of *N*-glycolylneuraminic (NeuGc) residues (Supplementary Table 1). In general, these oligosaccharides were detected only as minor peaks (in total < 3.5%).

#### *N*-Glycopeptide analysis

MALDI-MS analysis of HPLC fractions obtained from trypsin-digested TBEV E protein confirmed the occurrence of glycosylated peptides in the mass range 2532–4074 Da. The fraction with elution time 13–14 min was enriched with glycopeptides detected as [M + H]^+^ ions at *m/z* 2694, 3043, 3059, 3205 and 3246 (Fig. 2A). MS/MS spectra indicated presence of a peptide with *m/z* 1477.7 (Supplementary Fig. 3A) bearing oligosaccharides of the same compositions as analyzed on the same protein after digestion with PNGase F (Fig. 1). The glycopeptides detected in the fraction with elution time 16–18 min were observed in the range *m/z* 2993–4073 and again produced [M + H]^+^ ions with the characteristic mass differences of monosaccharide residues—146, 162 and 203 Da (Fig. 2B). In their MS/MS spectra, the total loss of monosaccharide residues was observed at *m/z* 1939.6 (Supplementary Fig. 3B); otherwise, the compositions of oligosaccharides were similar to those analyzed in the previous fraction. The exact determination of amino acid sequences was assigned after removal of glycans from the peptide backbone with PNGase F. It is known that asparagine (Asn or N), after losing its carbohydrate part, is modified to aspartic acid in the peptide (*N → D*). After deglycosylation, new peptides appeared at *m/z* 1478.6 and 1940.9 (Fig. 2C). Under tandem MS, these peaks produced fragment ions confirming peptide identities—TGDYVAA**D**ETTHSGR or VEPHTGDYVAA**D**ETTHSGR (Supplementary Fig. 4). Both sequences matched to the peptide described previously with the glycosylation site at Asn 154 in the class II membrane fusion protein TBEV E^34^ (inset in Fig. 2C).

**Figure 3:**
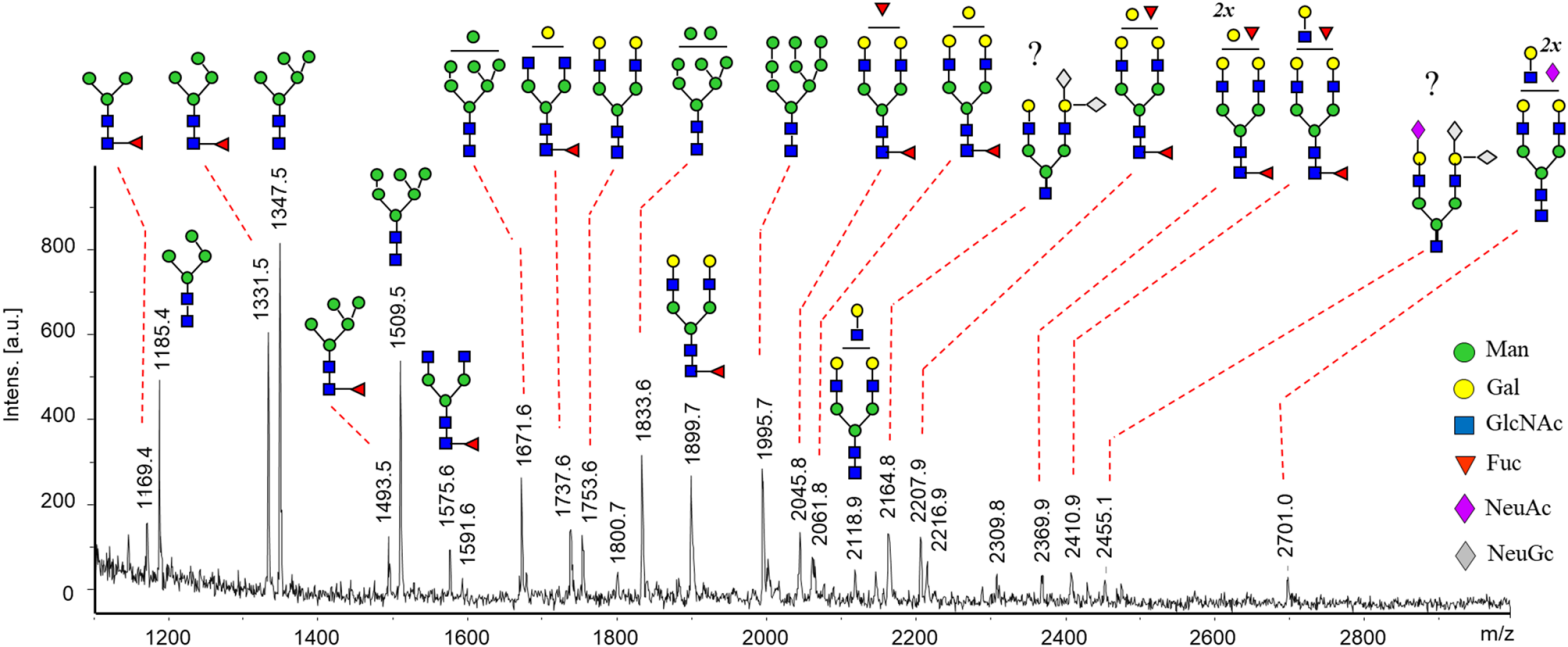
MALDI-MS spectrum of *N*-glycans recorded from TBEV grown in tick cells (IRE/CTVM19). Sample underwent incubation with PNGase F and neuraminidase, following SPE purification. Glycans are labeled at the reducing termini with PHN, and detected as MNa^+^ ions. Peaks with an asterisk correspond to sialylated glycans producing additional multiply sodiated ions and are the result of incomplete desialylation. For more detailed information about detected *N*-glycans see Supplementary Table 1.

**Figure 4:**
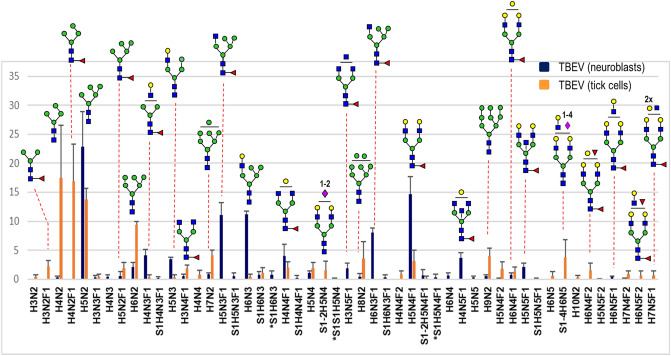
Graphical illustration of occurrence of *N*-glycans identified by MALDI-MS in the E glycoprotein of TBEV grown in human neuroblastoma (UKF-NB4; blue bars) and tick cells (IRE/CTVM19; orange bars). The graph was obtained from three experiments for each sample type and represents the averaged relative intensities with error bars as shown (± SD). *N-*glycan compositions are on the horizontal axis (H-hexose, N-GlcNAc, F-fucose, S-NeuAc, *S-NeuGc) and the calculated percentages (r.a) from measured peak intensities are on the vertical axis. For more information, see Supplementary Table 1.

### *N*-Glycan profile of TBEV grown in tick cells

The peaks corresponding to *N*-glycans isolated from virus grown in tick cells were detected with lower intensities than those in the sample from human cells. This reflects the slower TBEV replication and lower virus titers in these cells^27^. Nonetheless, MS analysis showed that the majority of the N-glycans were paucimannose with or without core fucose (*Man*_*4*_*GlcNAc*_*2*_* Fuc*_*0-1*_) and high-mannose (*Man*_*5-6*_*GlcNAc*_*2*_) glycans. Complex biantennary glycans with or without fucose were detected at *m/z* 1575.5, 1737.6, 1753.6, 1899.8 and those with additional fucose or galactose on the antenna at *m/z* 2045.9 and *m/z* 2061.8 (Fig. 3). The oligosaccharide peaks identified at *m/z* 2207.8, 2369.9 and 2410.9 indicated presence of both the above-mentioned monosaccharides residues. The other less-abundant glycan identified at *m/z* 2118.8 corresponded to the triantennary complex glycan with no fucose. This neutral structure was detected only after desialylation and originally was bearing one, two, three and four NeuAc residues (Supplementary Table 1). The peaks detected at *m/z* 2164.8 and 2455.1 also indicated presence of NeuGc residue/s. The graphical illustration of differences between the glycosylation patterns of TBEV E grown in human and tick cells is shown in Fig. 4.

### *N*-Glycans of human neuroblastoma cell line

The *N*-glycan profile of neuroblastoma cells showed minor presence of paucimannose and hybrid glycans. Oligomannose structures with compositions *Man*_*6-9*_*GlcNAc*_*2*_ and sialylated complex bi- and triantennary glycans were observed as major oligosaccharides (Supplementary Fig. 5). Among complex glycans, the dominant peaks were consistent with compositions of bi- and triantennary core fucosylated structures analyzed in desialylated pools at *m/z* 1899.7 and 2102.8 (*Gal*_*2*_*GlcNAc*_*2*_*-*_*3*_*Man*_*3*_*GlcNAc*_*2*_*Fuc*) or *m/z* 2264.9 (*Ga*_*3*_*GlcNAc*_*3*_*Man*_*3*_*GlcNAc*_*2*_*Fuc*_*1*_). The other significant peaks corresponded to the tetraantennary core fucosylated glycans and further extended with multiple lactosamine (LacNAc) residues (*Gal*_*4-8*_*GlcNAc*_*4–*__*8*_*Man*_*3*_*GlcNAc*_*2*_*Fuc*; *m/z* 2630.0, 2995.1, 3360.2, 3725.3, 4090.4). Peaks detected at *m/z* 2467.9. 2833.0, 3198.2 and 3563.3 indicated presence of the bisecting GlcNAc residue—*Gal*_*3-8*_*GlcNAc*_*3–*__*9*_*Man*_*3*_*GlcNAc*_*2*_*Fuc* (Supplementary Figs. 5 and 6).

### *N*-Glycans of tick cells

The analysis of tick cells showed significant presence of small (*Man*_*3-4*_*GlcNAc*_*2*_) and high-mannose (*Man*_*5-9*_*GlcNAc*_*2*_) glycans, including their fucosylated structures (*Man*_*3-5*_*GlcNAc*_*2*_*Fuc*). These glycans dominated in the spectra recorded from cells grown either under standard conditions or in the medium without FBS (Supplementary Fig. 7). The other abundant glycans corresponded to the complex bi- and triantennary sialylated forms with one, two, three and four NeuAc residues (*m/z* 2792.4, 2880.4, 3241.6 and 3602.8 when the sample was analyzed after permethylation). These glycans after desialylation and derivatization with PHN produced peaks at *m/z* 1753.6 (*Gal*_*2*_*GlcNAc*_*2*_*Man*_*3*_*GlcNAc*_*2*_*)* and *m/z* 2118.8 (*Gal*_*3*_*GlcNAc*_*3*_*Man*_*3*_*GlcNAc*_*2*_, triantennary glycan with branching on the 3-arm) (Supplementary Table 1). Among complex core fucosylated glycans, only the biantennary structure having additional fucose and galactose residues on one of the antennae (*Gal*_*3*_*Fuc*_*1*_*GlcNAc*_*2*_*Man*_*3*_*GlcNAc*_*2*_*Fuc)* was detected with highest abundance. Hybrid glycans were detected with lower abundances and showed presence of NeuAc residue (*m/z* 2156.1 and 2248.3; Supplementary Fig. 7). MS spectra recorded from tick cell samples grown in medium without FBS showed presence of the same glycans as mentioned above with one main difference; the proportion of sialylated triantennary complex *N*-glycans was reduced (Supplementary Fig. 7B).

## Discussion

*N*-Glycans on viral proteins play essential roles in replication, immunogenicity and pathogenicity^35^. Previous studies on flaviviruses, such as dengue, West Nile and Zika viruses, demonstrated that *N*-glycosylation of the E protein is a critical determinant of viral pathogenesis in both mammalian hosts and mosquito vectors^16,36–38^. In the case of TBEV, *N*-glycosylation plays an important role in proper conformation of the E protein during secretion in mammalian cells^10,14^. On the other hand, the TBEV secretory/maturation process in tick cells seems to be independent of E protein *N*-glycosyslation^14^. In our study, observed dissimilarities in profiles and preferences in occurrence of different *N*-glycan structures detected in TBEV E grown in human and tick cells are obvious. These discrepancies reflect employment of different glycosyltransferases depending on the host cell. To validate these significant variations and confirm the originality in both profiles, we also scrutinized *N*-glycans occurring in uninfected human neuroblastoma and tick cells. A comparison of profiles analyzed in all four samples is summarized in Supplementary Fig. 8.

E glycoprotein obtained from TBEV grown in neuroblastoma cells showed a unique profile as follows. The glycan with five mannose residues—*Man*_*5*_*GlcNAc*_*2*_ (*m/z* 1347) was detected as the major oligosaccharide in infected neuronal cells (Fig. 1). The other oligomannose structures bearing 6–9 mannose residues were observed only as minor peaks (< 4%). The second dominant glycan in the spectrum corresponded to the neutral biantennary core fucosylated structure—*Gal*_*2*_*GlcNAc*_*2*_*Man*_*3*_*GlcNAc*_*2*_*Fuc *(*m/z* 1899)*.* The other complex core fucosylated glycans observed with lower abundances were carrying one, two or three non-substituted GlcNAc residues (*m/z* 1737, 1778, 1940 and 2102). The third main structures represented were a group of hybrid glycans with compositions *Gal*_*0-1*_*GlcNAc*_*1*_*Man*_*3-5*_*GlcNAc*_*2*_*Fuc*_*0-1*_. Sialylated glycans were generally present at low abundance.

The glycan profile of E glycoprotein from TBEV grown in neuroblastoma cells was similar to that obtained through analysis of tryptic glycopeptides (Fig. 2). The data provided additional information about the glycosylation site in TBEV E protein at Asn 154. Our observation is consistent with previously-published findings that TBEV E protein has only a single *N*-linked glycosylation site^10^.

On the other hand, the glycan profile of TBEV grown in tick cells showed a significantly different pattern to the profile of E glycoprotein isolated from infected neuroblastoma cells. In contrast to virus grown in mammalian cells, paucimannose (*Man*_*3-4*_*GlcNAc*_*2*_*Fuc*_*0-1*_) and high-mannose (*Man*_*5-9*_*GlcNAc*_*2*_) types were the major glycans detected in virus grown in tick cells. Complex biantennary glycans were mostly core fucosylated. While no glycan with fucose(s) on the antenna was observed in the profile of E protein from TBEV grown in neuroblastoma cells, in TBEV from tick cells these glycans were detected e.g. at *m/z* 2045.8, 2207.8. The other difference was in the absence of hybrid glycans. Sialylated oligosaccharides were also observed with lower abundances and the *m/z* data indicated presence of both NeuAc and NeuGc residues. Although the low concentration of sample precluded a more detailed structural examination, especially of glycans with low abundances, the differences between the profiles of the two infected samples are evident.

MS analysis of uninfected neuroblastoma cells confirmed the different pattern in the *N-*glycan profile of TBEV grown in these cells. Here, the main glycans corresponded to high-mannose structures (*Man*_*6-9*_*GlcNAc*_*2*_) and sialylated bi, tri and tetraantennary complex core fucosylated glycans with/without bisecting moiety which were detected in desialylated pools at *m/z* 1899.7, 2102.8, 2264.9, 2467.9, 2630.0 and 2833.0 (*Gal*_*2-4*_*GlcNAc*_*2–*__*5*_*Man*_*3*_*GlcNAc*_*2*_*Fuc*). These glycans were extended with additional LacNAc residue(s) (*Gal*_*5-8*_*GlcNAc*_*5–*__*8*_*Man*_*3*_*GlcNAc*_*2*_*Fuc*; *m/z* 2995.1, 3198.2, 3360.2, 3563.3, 3725.3). It should be noted that none of the tetraantennary or higher glycans were found in any of other three samples analyzed here (Supplementary Fig. 8).

Further investigation of *N*-glycans from uninfected tick cells showed a close resemblance to the pattern of TBEV grown in these cells, in the presence of paucimannose and high-mannose structures. However, the most notable differences were in the higher proportion of sialylated complex and hybrid glycans. It was hypothesized that the presence of sialylated oligosaccharides in tick cells could be a consequence of binding the sialylated species from the FBS in the culture medium^39^. However, in our experiments very similar profiles were recorded from tick cells when cultured with and without FBS. The decline was detected mainly in the case of the complex triantennary sialylated glycan *NeuAc*_*1-4*_*Gal*_*3*_*GlcNAc*_*3*_*Man*_*3*_*GlcNAc*_*2*_. A similar trend was observed in the case of uninfected neuroblastoma cells. In desialylated pools this glycan was detected as an abundant peak at *m/z* 2118 (*Gal*_*3*_*Fuc*_*1*_*GlcNAc*_*2*_*Man*_*3*_*GlcNAc*_*2*_) and showed a steep decline when the cells were grown without FBS.

In conclusion, in this study we provide comprehensive mapping of the glycosylation on the TBEV E glycoprotein when the same virus type was grown in human and tick cells. We validated the originalities in profiles by the examination of *N*-glycans isolated from uninfected host cells. The results reported here expand existing crystallography data on the E glycoprotein. Furthermore, the data provide evidence that the *N*-glycan profile of TBEV E glycoprotein depends strongly on the host cell type. The results presented in this study may contribute to understanding of TBEV biology in mammals and ticks. As proteins that bind to *N*-linked glycans of enveloped viruses represent a new emerging class of highly effective antivirals, our results might be useful in research for development of carbohydrate-binding agents as potential inhibitors of TBEV.

## Supplementary information

Supplementary Information.

## Data Availability

The datasets used and/or analyzed during the current study are available from the corresponding author on reasonable request.
