# Supplementary material for: Comprehensive N-glycosylation mapping of envelope glycoprotein from tick-borne encephalitis virus grown in human and tick cells
Source: Sci Rep. 2020 Aug 6;10:13204. doi: 10.1038/s41598-020-70082-2 (PMC7411051; doi:10.1038/s41598-020-70082-2)
Supplement: Supplementary file 1 — Supplementary Information. [file 41598_2020_70082_MOESM1_ESM.pdf]

SUPPLEMENTARY INFORMATION

for

Comprehensive *N*-glycosylation mapping of envelope glycoprotein from tick-borne encephalitis virus grown in human and tick cells

Erika Lattová [a*], Petra Straková [b], Petra Pokorná-Formanová [b], Libor Grubhoffer [c], Lesley Bell-Sakyi [d], Zbyněk Zdráhal [a,e], Martin Palus [b,c], Daniel Ruzek [b,c*]

[a] *Central European Institute for Technology, Masaryk University, Kamenice 5, CZ-62500 Brno, Czech Republic*

[b] *Veterinary Research Institute, Hudcova 296/70, CZ-62100 Brno, Czech Republic*

[c] *Institute of Parasitology, Biology Centre of the Czech Academy of Sciences, Branisovska 31, CZ-37005 Ceske Budejovice, Czech Republic*

[d] *Department of Infection Biology and Microbiome, Institute of Infection, Ecological and Veterinary Sciences, University of Liverpool, 146 Brownlow Hill, Liverpool, L3 5RF, UK*

[e] *National Centre for Biomolecular Research, Faculty of Science, Masaryk University, Kamenice 5, CZ-62500 Brno, Czech Republic*

**Correspondence:*
Erika Lattová (erika.lattova@gmail.com) or Daniel Ruzek (ruzekd@paru.cas.cz)

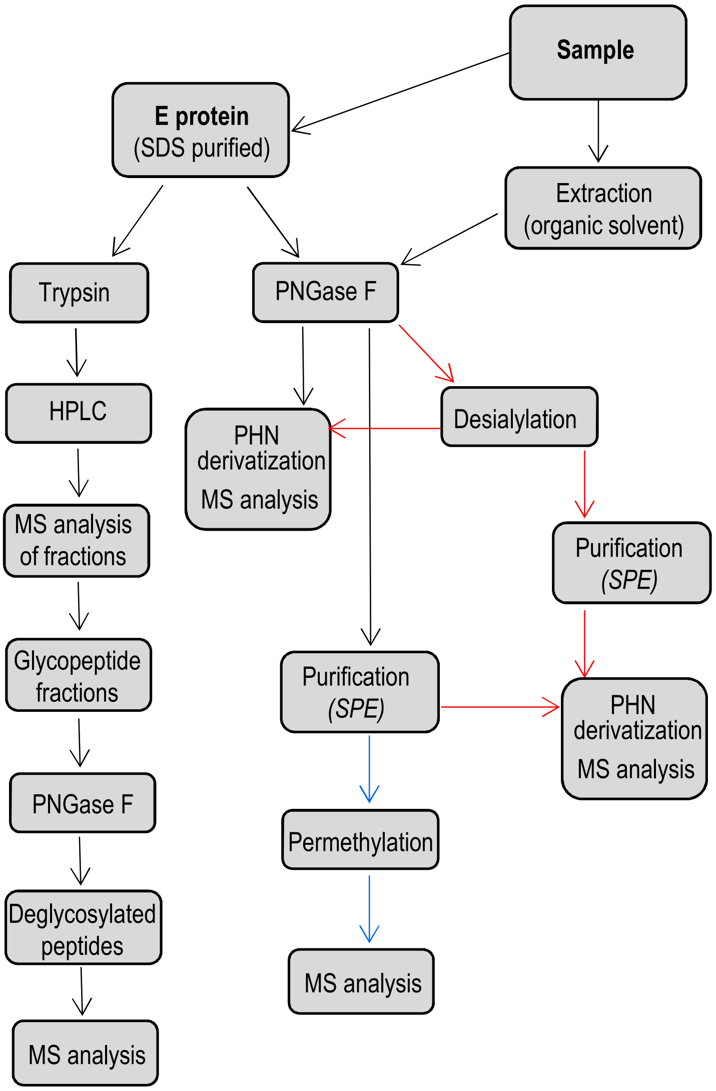

Supplementary Scheme 1. A simple flowchart depicting the applying of preparation methods for investigation of *N*-glycosylation in samples analyzed in this study.

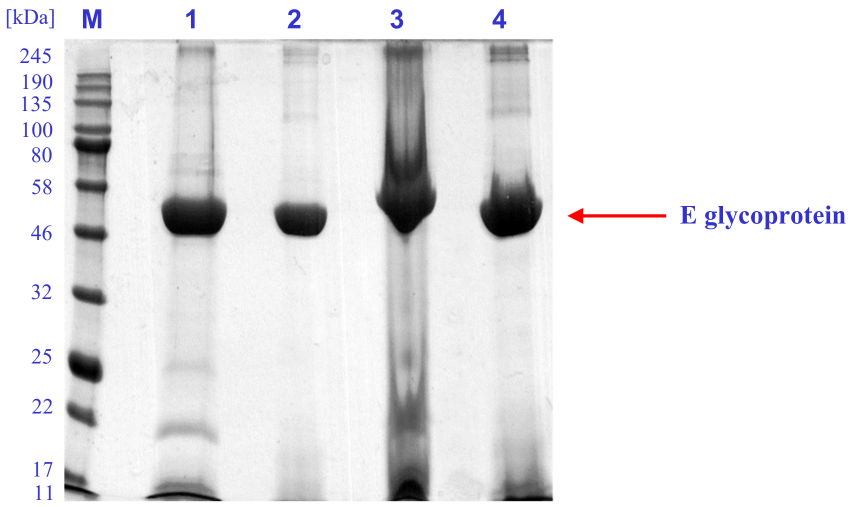

Supplementary Figure 1. SDS-Page electrophoretic purification of the envelope E glycoprotein obtained from TBEV grown in human neuroblastoma cells. The samples were dissolved in 50mM Tris-HCl (pH=6.8) and mixed for 1 h. The 1-DE electrophoresis was performed on 10% SDS using Bio-Rad equipment (Protean II xi Cell; 20 min at 100 V and 50 min at 200 V). Gel was stained with Bio-Safe Coomassie Stain (Bio-Rad). **M** - proteins standard ladder; line **1** - 5ul load of TBEV sample in loading buffer with DTT; line **2** - 5ul of TBEV sample in loading buffer without DTT; line **3 -** 10ul load of TBEV in loading buffer with DTT; line **4 -** 10ul load of TBEV in loading buffer without DTT.

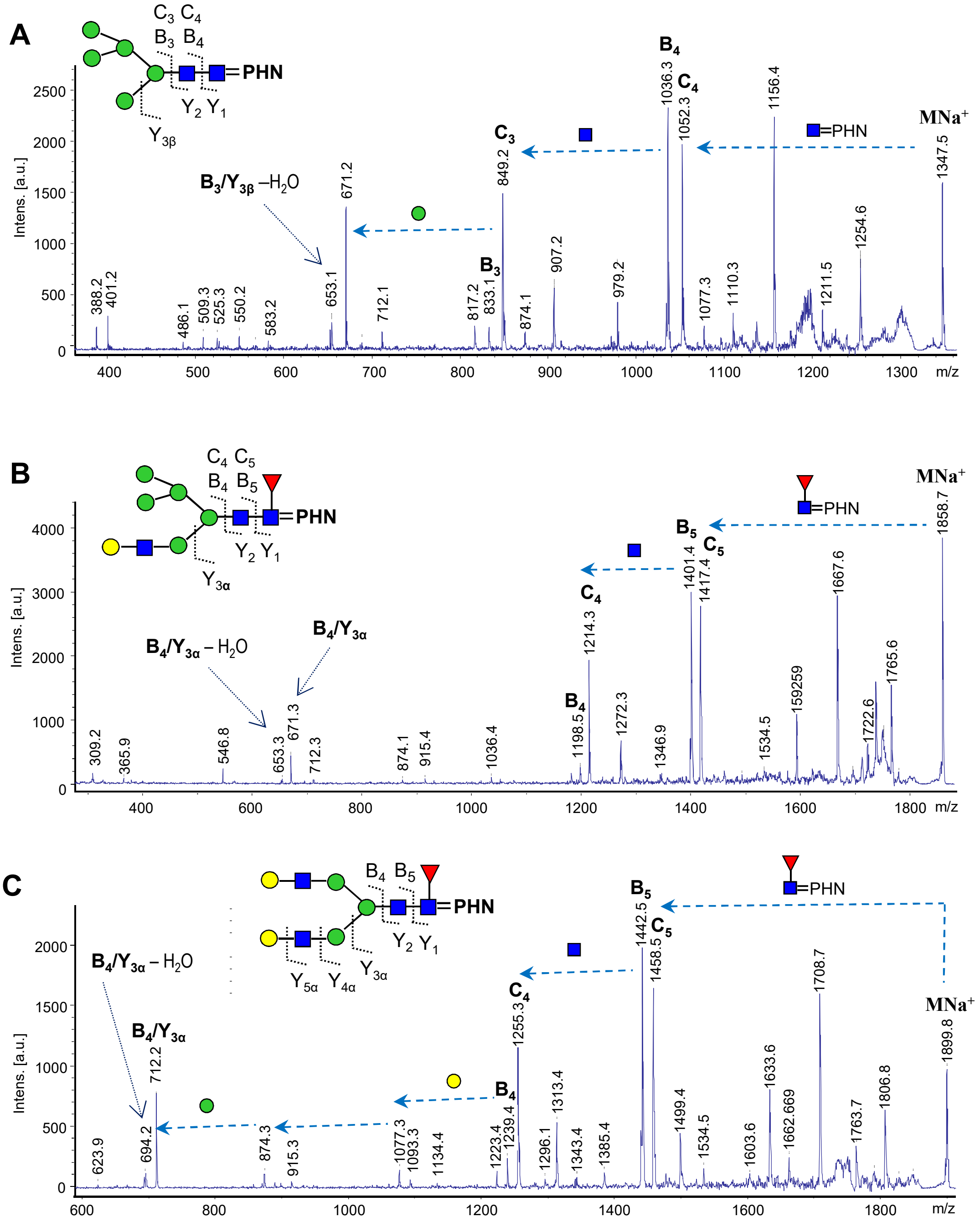

Supplementary Figure 2. MALDI-MS/MS spectra of *N*-glycans with precursor ions at *m/z*: **A**) 1347.5; **B**) 1858.6; and **C**) 1899.8. Spectra were recorded from PNGase F digest of TBEV E glycoprotein obtained from virus grown in human neuroblastoma cells. Glycans are derivatized with phenylhydrazine (PHN) at the reducing termini. All ions are sodiated. Fragmentation schemes represent dominant isomeric structures suggested on the basis of the presence of assigned diagnostically useful fragment ions (*Ref. 31*).

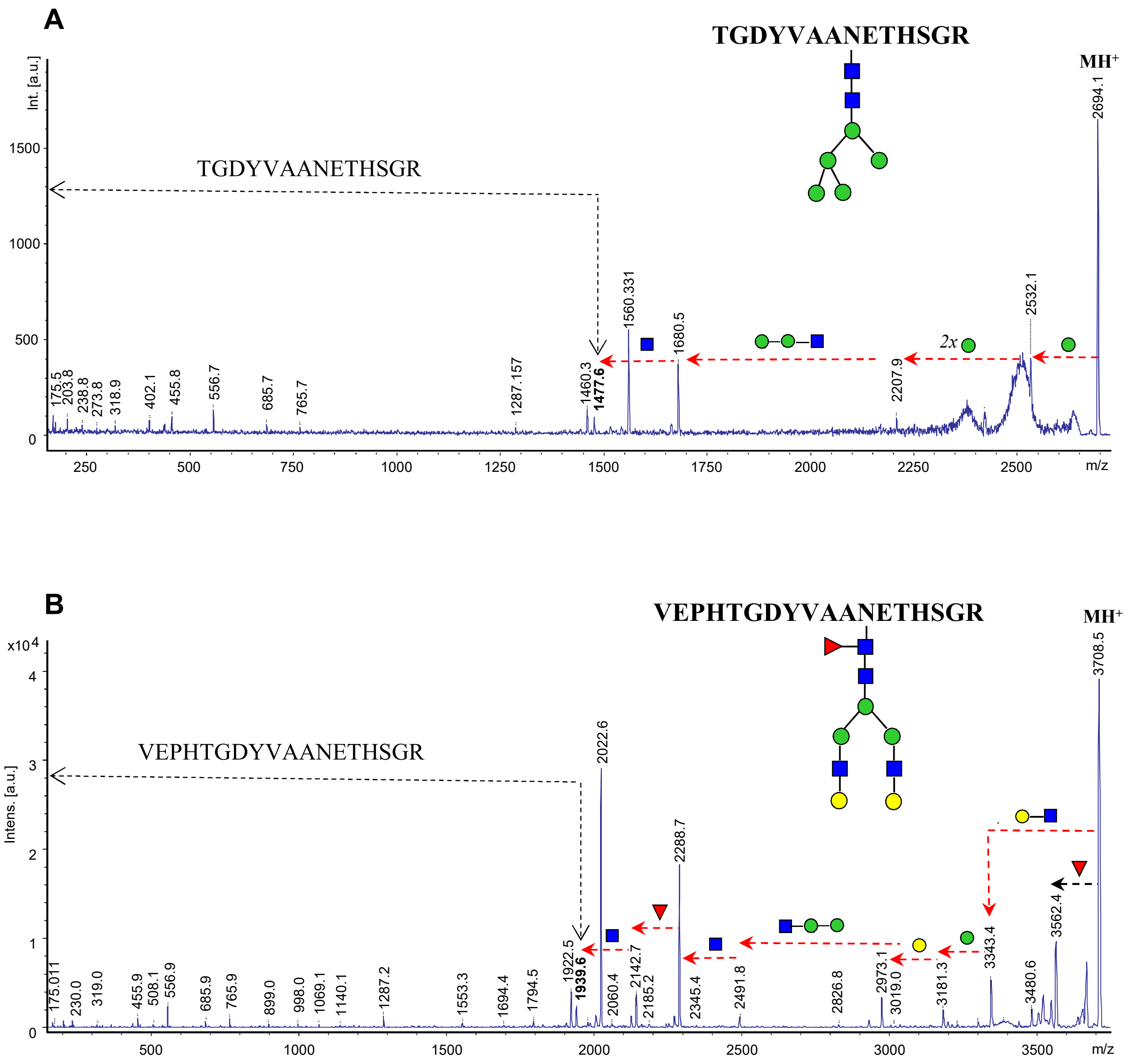

Supplementary Figure 3. MALDI-MS/MS spectra of glycopeptides obtained from the trypsin digested TBEV E glycoprotein and recorded for precursor ions at *m/z*: **A**) 2694.1; and **B**) 3708.5. Oligosaccharide structures were assigned based on MS/MS analysis of *N*-glycans cleaved from the same glycoprotein with PNGase F (e.g. presented in Supp. Fig. 2). For more information about the exact assignment of amino acid sequences detected in glycopeptides see the following Supp. Fig. 4.

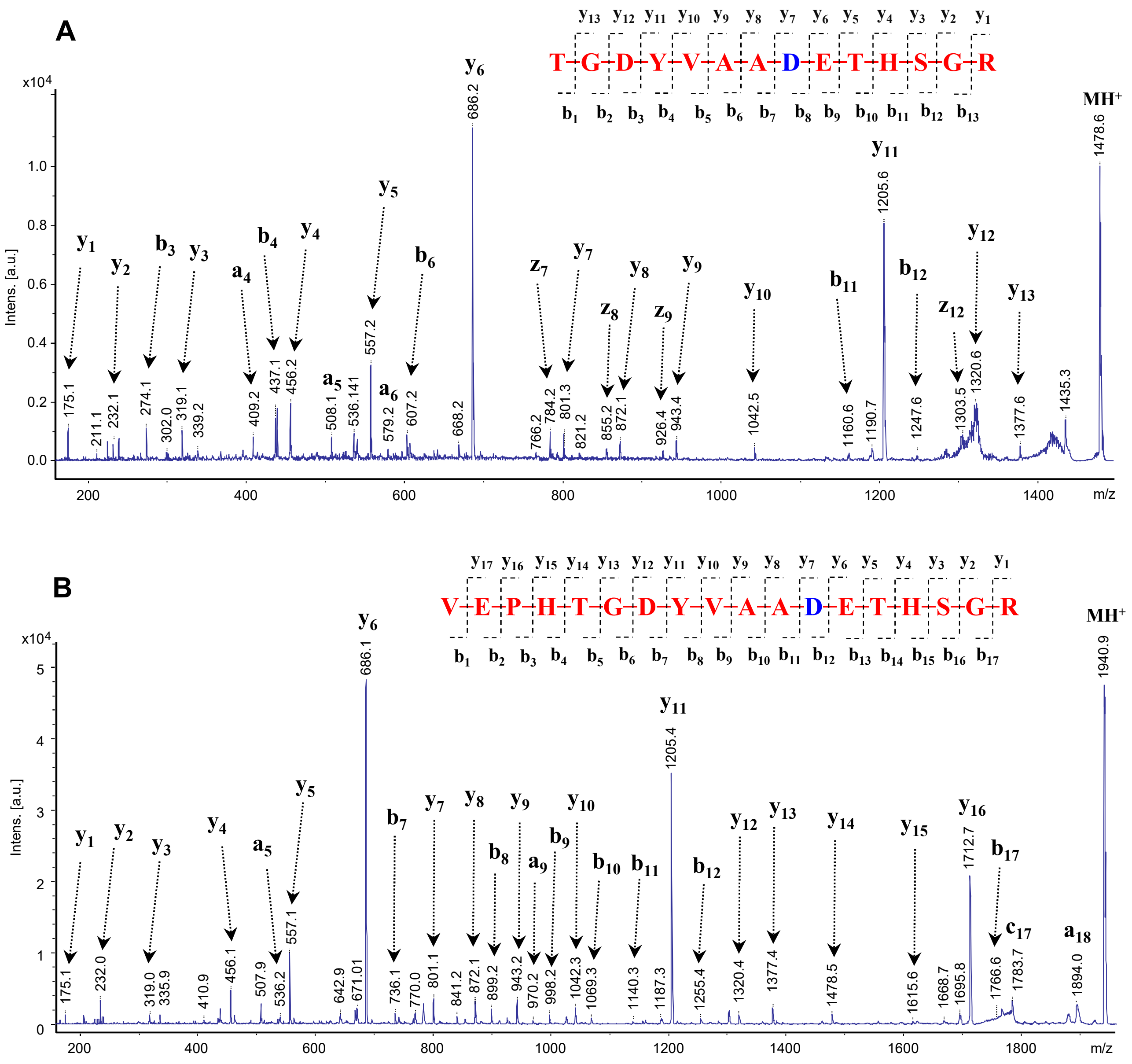

Supplementary Figure 4. MALDI-MS/MS spectra of deglycosylated peptides shown in Fig. 2C with *m/z* precursor ions: **A**) 1478.6 and **B**) 1940.9. All ions are as MH^+. The fragment ions are annotated according to nomenclature proposed by *Roepstorf & Fohlman (Ref.*.33).

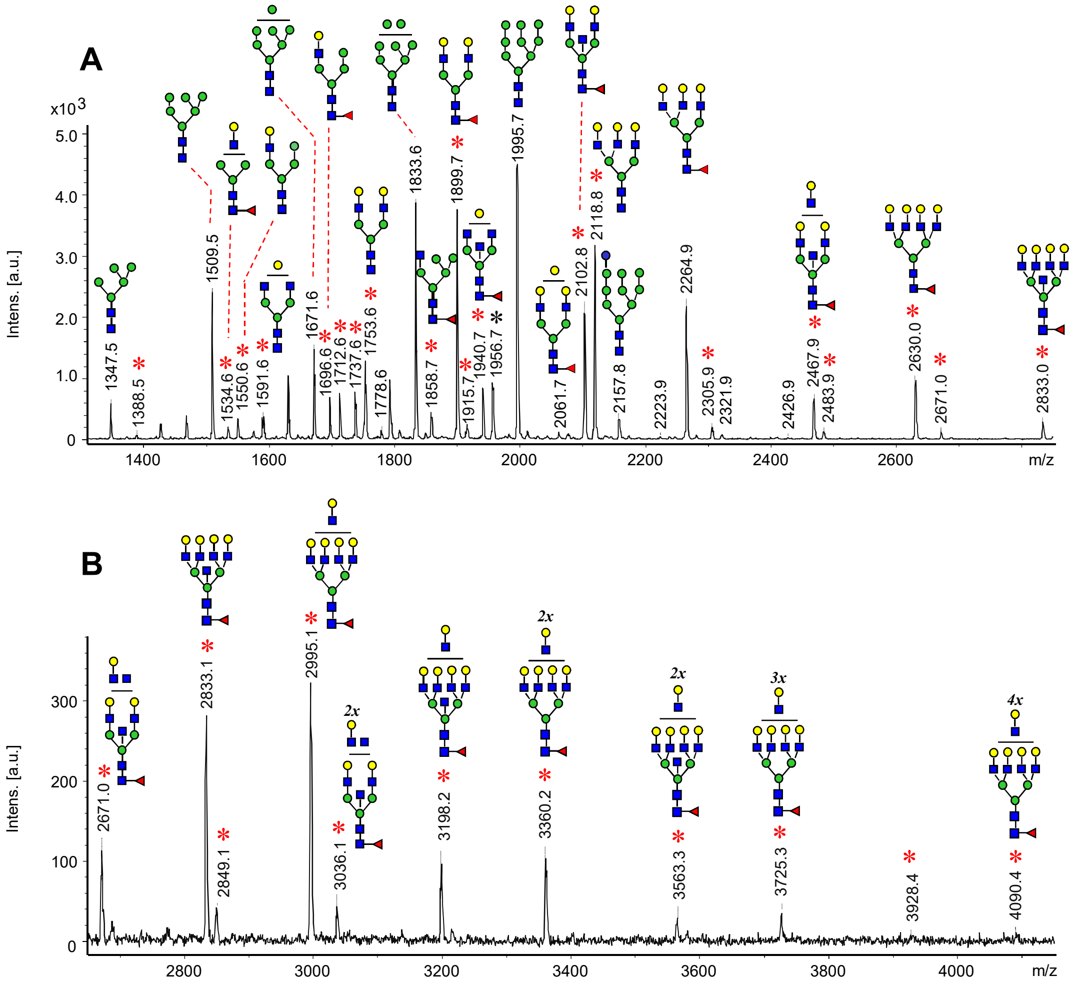

Supplementary Figure 5. MALDI-MS spectrum of *N*-glycans recorded from uninfected human neuroblastoma cells (UKF-NB4; cultured in the presence of FBS) after incubation with PNGase F and neuraminidase. In **A** are glycans with *m/z* between 1310 – 2850; and in **B** with *m/z* 2650 – 4150. Glycans are labeled with PHN at the reducing termini and detected as MNa^+ ions. Peaks with no assigned *m/z* values correspond to the compositions of oligohexoses. The peaks with red asterisks were not detected before incubation with neuraminidase indicating that these glycans were originally sialylated. For details in regard to structural assignments see Supp. Fig. 6.

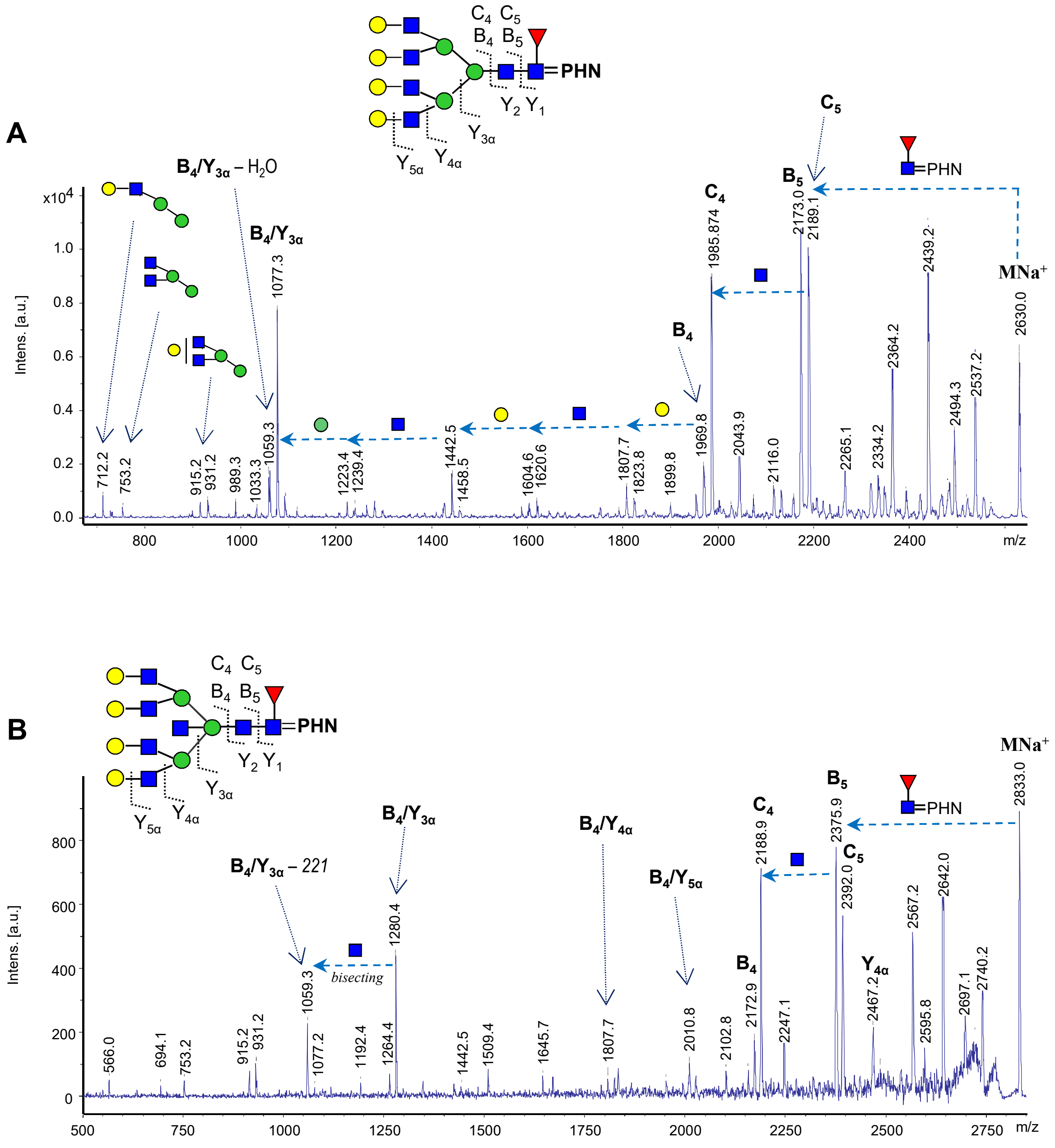

Supplementary Figure 6. Selected MALDI-MS/MS spectra of *N*-glycans recorded for the precursor ions with *m/z*: **A**) 2630.0 and **B**) 2833.0. Glycans were obtained from uninfected human neuroblastoma cells (UKF-NB4) after incubation with PNGase F and neuraminidase, and labeled with PHN at the reducing termini. All ions are sodiated. Fragmentation schemes represent dominant isomeric structures suggested on the presence of assigned diagnostically useful fragment ions (*Ref. 31*).

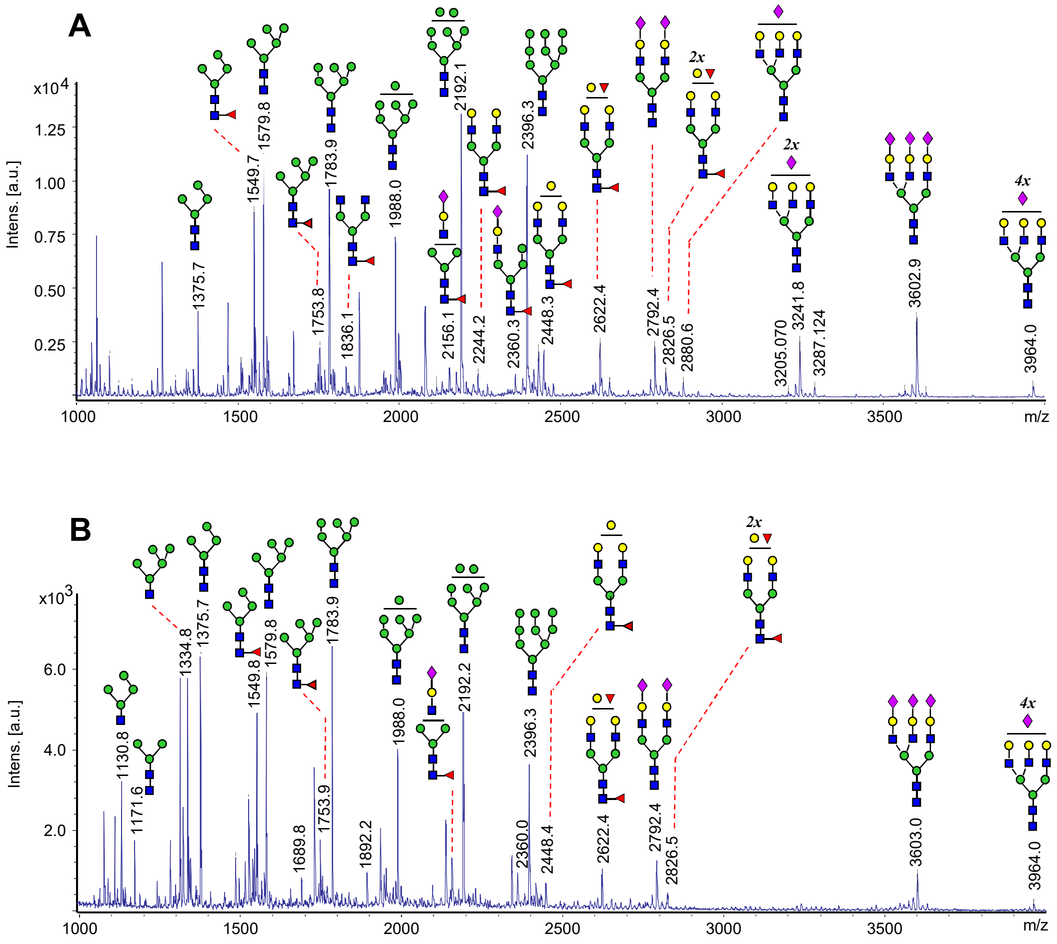

Supplementary Figure 7. MALDI-MS spectra of permethylated *N*-glycans obtained after incubation with PNGase F and SPE purification from uninfected tick cells cultured: **A**) in the presence of FBS; and **B**) in serum-free medium. Glycan peaks are detected as MNa$^+$ ions. Unlabeled peaks are consistent with compositions of oligohexoses.

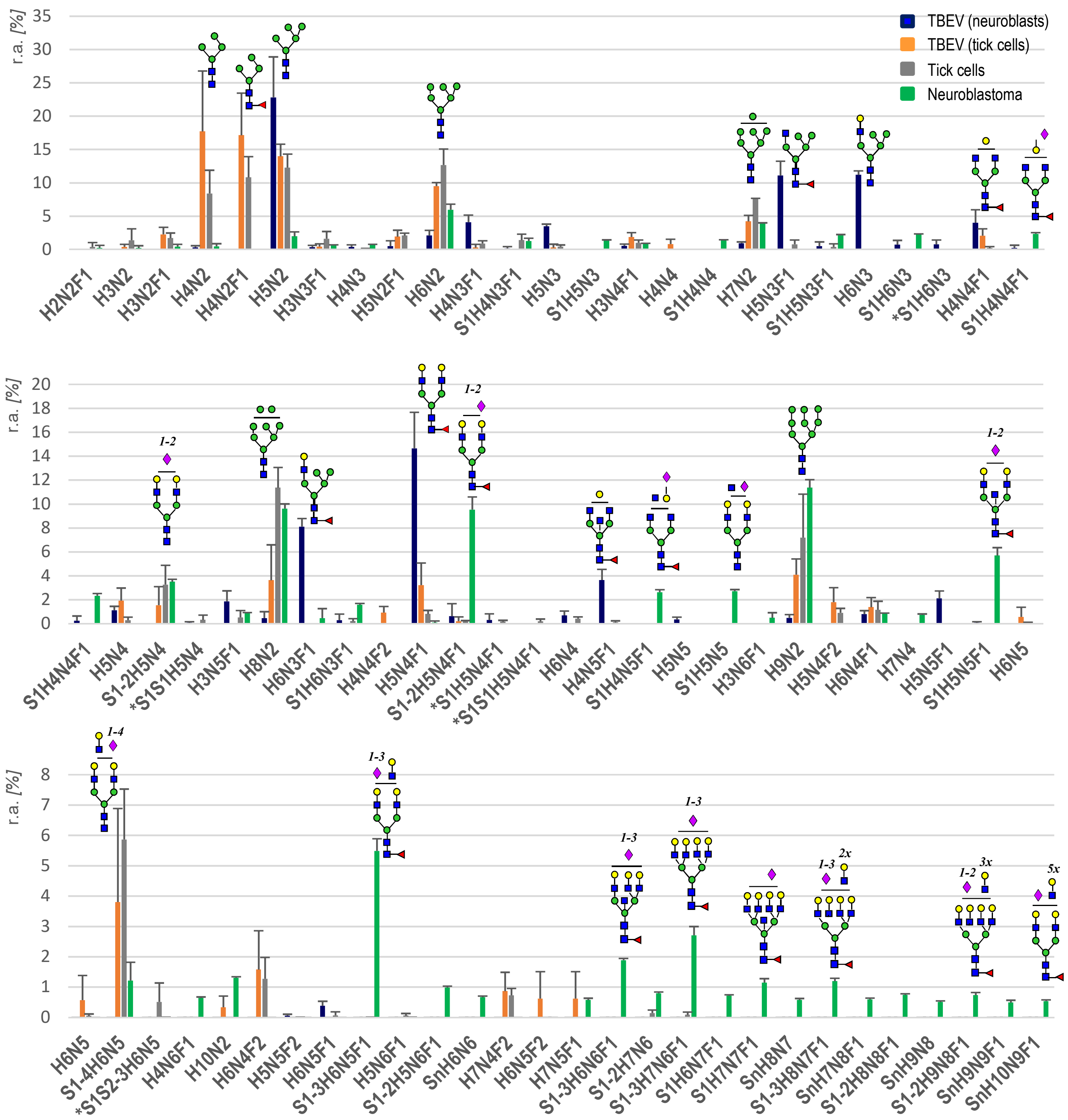

Supplementary Figure 8. Graphical illustration of occurrence of *N*-glycans identified by MALDI-MS in the E glycoprotein of TBEV grown in human neuroblastoma (blue) and tick cells (orange) versus uninfected tick (grey) and neuroblastoma (green) cells cultured in serum-free medium. The graphs were obtained from 3 experiments for each sample type and represent the averaged relative intensities with error bars as shown (±SD). *N*-glycan compositions are on the horizontal axis and their percentage abundances are depicted on the vertical axis. H-hexose, N-GlcNAc, F-fucose, S-NeuAc, *S-NeuGc, Sn-sialylation confirmed only after incubation with neuraminidase.

Supplementary Table 1. The list of *N*-glycans identified by MALDI-MS on the surface of tick-borne encephalitis virus derived from human neuronal (**A**) and tick cells (**B**), and uninfected neuroblastoma (**C**) and tick (**D**) cells.

Structure	Composition	m/z		Sample			
		**PHN** *[MNa]*$^+$	**PE** *[MNa]*$^+$	**A**	**B**	**C**	**D**
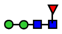	H2N2F1	1007.38	1141.57	ND	ND	+	+
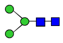	H3N2	1023.37	1171.58	ND	+	+	+
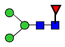	H3N2F1	1169.43	1345.67	ND	+	+	+
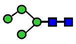	H4N2	1185.43	1375.68	+	+++	+	+++
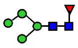	H4N2F1	1331.48	1549.77	ND	+++	ND	+++
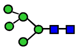	H5N2	1347.48	1579.78	+++	+++	+	+++
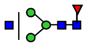	H3N3F1	1372.51	1590.80	+	+	+	+
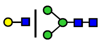	H4N3	1388.51	1620.81	+	ND	+	+
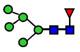	H5N2F1	1493.54	1753.87	+	+	ND	+
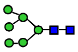	H6N2	1509.53	1783.88	+	+++	++	+++
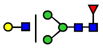	H4N3F1	1534.56	1794.90	++	+	ND	+
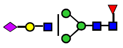	S1H4N3F1	1825.66	2156.07	+	ND	+	+
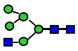	H5N3	1550.56	1824.91	++	+	ND	+
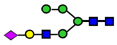	S1H5N3	1841.64	2186.05	ND	ND	+	ND
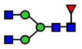	H3N4F1	1575.59	1835.93	+	+	+	+
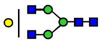	H4N4	1591.59	1865.94	ND	ND	ND	+
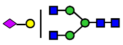	S1H4N4	1882.68	2227.11	ND	ND	+	ND
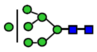	H7N2	1671.58	1987.98	+	++	++	++
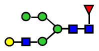	H5N3F1	1696.62	1998.99	+++	ND	ND	+
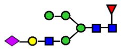	S1H5N3F1	1987.75	2360.16	+	ND	+	+
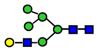	H6N3	1712.62	2029.01	+++	ND	ND	ND
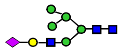	S1H6N3	2003.72	2390.18	+	ND	+	ND
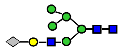	*S1H6N3	2019.70	2420.19	+	ND	ND	ND

continues

Structure	Composition	m/z		Sample			
		PHN *$[MNa]^+$*	PE *$[MNa]^+$*	A	B	C	D
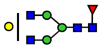	H4N4F1	1737.65	2040.03	++	+	ND	+
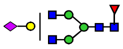	S1H4N4F1	2028.75	2401.20	+	ND	++	ND
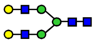	H5N4	1753.64	2070.04	+	+	ND	+
1-2 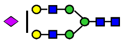	S1-2H5N4	2044.74 (S1)<br>2335.83 (S2)	2431.21 (S1)<br>2792.38 (S2)	ND	+	+	+
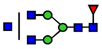	H3N5F1	1778.68	2081.05	++	ND	+	+
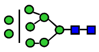	H8N2	1833.64	2192.08	+	++	+++	+++
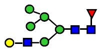	H6N3F1	1858.68	2203.10	++	ND	+	ND
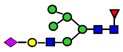	S1H6N3F1	2149.78	2564.27	+	ND	+	+
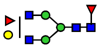	H4N4F2	1883.70	2214.11	ND	+	ND	ND
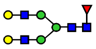	H5N4F1	1899.69	2244.13	+++	++	+	+
1-2 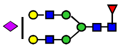	S1-2H5N4F1	2190.79 (S1)<br>2481.89 (S2)	2605.30 (S1)<br>2966.48 (S2)	+	+	+++	+
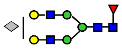	*S1H5N4F1	2206.79	2635.31	+	ND	ND	+
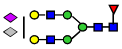	*S1S1H5N4F1	2497.88	2996.48	ND	ND	ND	+
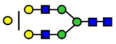	H6N4	1915.70	2274.14	+	ND	ND	+
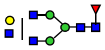	H4N5F1	1940.72	2285.15	+	ND	ND	+
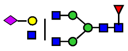	S1H4N5F1	2231.82	2646.32	ND	ND	++	ND
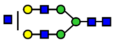	H5N5	1956.72	2315.16	+	ND	ND	ND
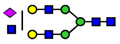	S1H5N5	2247.81	2676.33	ND	ND	++	ND
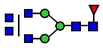	H3N6F1	1981.75	2326.18	ND	ND	+	ND
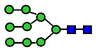	H9N2	1995.69	2396.18	+	++	+++	+++
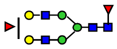	H5N4F2	2045.75	2418.21	ND	+	ND	+

continues

Structure	Composition	m/z		Sample			
		PHN *[MNa]⁺*	PE *[MNa]⁺*	A	B	C	D
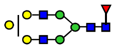	H6N4F1	2061.75	2448.23	+	+	+	+
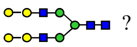	H7N4	2077.74	–	ND	ND	+	ND
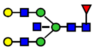	H5N5F1	2102.78	2489.25	++	ND	ND	ND
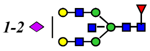	S1H5N5F1	2393.88	2850.43 (S1)<br>3211.60 (S2)	ND	ND	++	ND
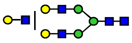	H6N5	2118.77	2519.26	ND	ND	ND	+
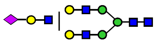	S1H6N5	2409.87	2880.43	ND	+	+	+
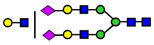	S2H6N5	2700.96	3241.60	ND	+	+	++
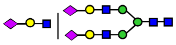	S3H6N5	2992.06	3602.78	ND	+	+	+
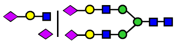	S4H6N5	3283.16	3963.95	ND	+	ND	+
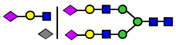	*S1S3H6N5	–	3993.96	ND	ND	ND	+
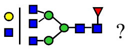	H4N6F1	2143.80	2530.29	ND	ND	+	ND
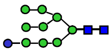	H10N2	2157.74	2600.28	ND	+	+	ND
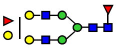	H6N4F2	2207.81	2622.31	ND	+	ND	+
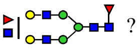	H5N5F2	2248.83	2663.34	+	ND	ND	ND
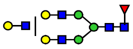	H6N5F1	2264.83	2693.35	+	ND	ND	+
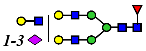	S1-3H6N5F1	2555.93 (S1)	3054.53 (S1)<br>3415.70 (S2)<br>3776.88 (S3)	ND	ND	+++	ND
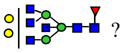	H5N6F1	2305.85	2734.38	ND	ND	+	+
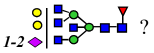	S1-2H5N6F1	2305.85*	3095.55 (S1)<br>3456.73 (S2)	ND	ND	+	ND
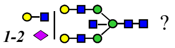	S1-2H6N6	2321.85*	–	ND	ND	+	ND
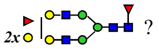	H7N4F2	2369.86	2826.47	ND	+	ND	+
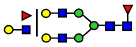	H6N5F2	2410.89	–	ND	+	ND	ND

continues

Structure	Composition	m/z		Sample			
		PHN *[MNa]*+	PE *[MNa]*+	A	B	C	D
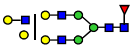 ?	H7N5F1	2426.88	–	ND	+	+	ND
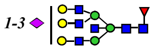	S1-3H6N6F1	2467.91*	3299.65 (S1)<br>3660.82 (S2)<br>4021.99 (S3)	ND	ND	++	ND
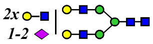	S1-2H7N6	2483.90*	3329.67 (S1)<br>3690.84 (S2)	ND	ND	+	ND
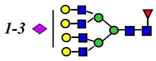	S1-4H7N6F1	2629.96*	3503.74 (S1)<br>3864.92 (S2)<br>4226.10 (S3)	ND	ND	++	ND
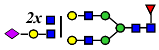 ?	S1H6N7F1	2670.99*	3544.78 (S1)	ND	ND	+	ND
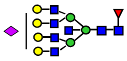	S1H7N7F1	2833.04*	3748.87 (S1)	ND	ND	+	ND
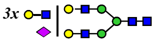 ?	SnH8N7	2849.03*	–	ND	ND	+	ND
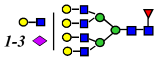	S1-3H8N7F1	2995.09*	3952.98 (S1)<br>4314.16 (S2)<br>4675.33 (S3)	ND	ND	+	ND
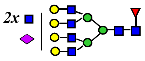 ?	SnH7N8F1	3036.12*	–	ND	ND	+	ND
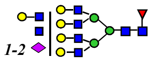 ?	S1-2H8N8F1	3198.17*	4198.10 (S1)<br>4560.29 (S2)	ND	ND	+	ND
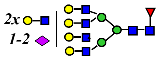	S1-2H9N8F1	3360.22*	4402.20 (S1)<br>4763.38 (S2)	ND	ND	+	ND
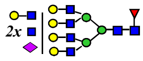 ?	SnH8N9F1	3401.25*	–	ND	ND	+	ND
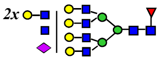 ?	SnH9N9F1	3563.30*	–	ND	ND	+	ND
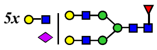 ?	SnH10N9F1	3725.36*	–	ND	ND	+	ND
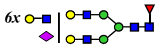 ?	SnH11N10F1	4090.43*	–	ND	ND	+	ND

N-Glycan compositions and structures were derived from molecular masses, MS/MS fragmentations *(Ref. 31)* and exoglycosidase cleavages. Suggested structures represent dominant isomers identified for each glycan composition. Key symbols: H-*hexose*, N-*GlcNAc*, F-*Fuc*, S-*NeuAc*, *S-*NeuGc*; red triangl-*Fuc*, blue square-*GlcNAc*, green circle-*Man*, yellow circle-*Gal*, violet diamond-*NeuAc*, grey diamond-*NeuGc*.

* Sialylation confirmed only after incubation with neuraminidase (red asterisk).

? Glycan peak not sufficient for structural analysis;

– Glycan peak was not observed in phenylhydrazine (PHN) derivatized or permethylated (PE) pools;

ND = not detected at all; **+** minor glycan; **+++** major glycan
